# Supplementary material for: Novel In Vitro Models for Cell Differentiation and Drug Transport Studies of the Human Intestine
Source: Cells. 2023 Sep 27;12(19):2371. doi: 10.3390/cells12192371 (PMC10572004; doi:10.3390/cells12192371)
Supplement: Supplementary file 1 [file cells-12-02371-s001.zip › cells-2607006-supplementary.docx]

Supplementary Materials


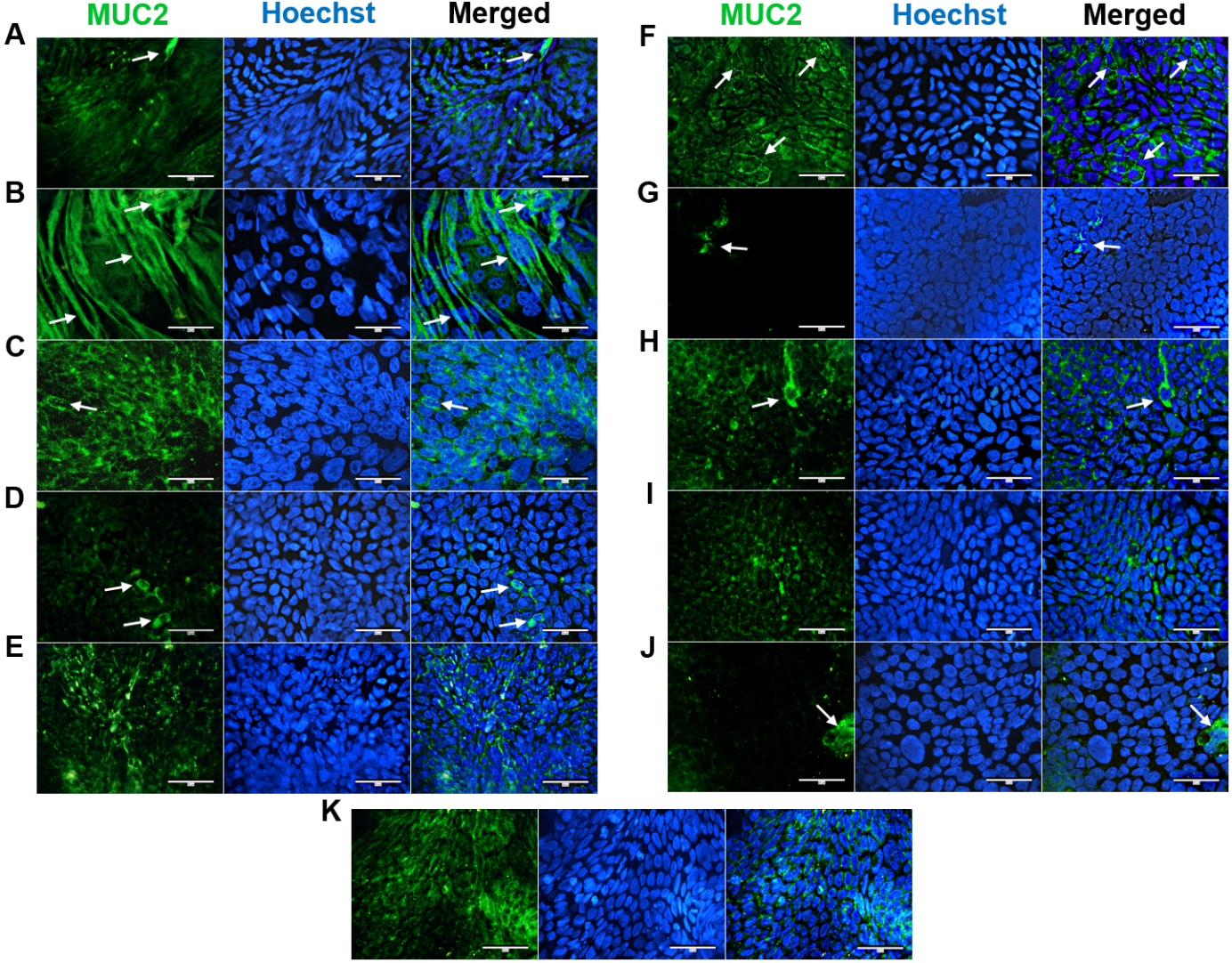


**Figure S1.** Immunofluorescence analysis of MUC-2 protein localization. (**A**) HROC32, (**B**) HROC43, (**C**) HROC60, (**D**) HROC80 T1 M1, (**E**) HROC126, (**F**) HROC159 T2 M4, (**G**) HROC183 T0 M2, (**H**) HROC217 T1 M2, (**I**) HROC239 T0 M1, (**J**) HROC383 and (**K**) Caco-2. Cells were stained for localization of MUC-2 by direct immunofluorescence. Arrowheads illustrate representative areas of significant fluorescent staining patterns indicative of GCs. Bars = 50 µm.


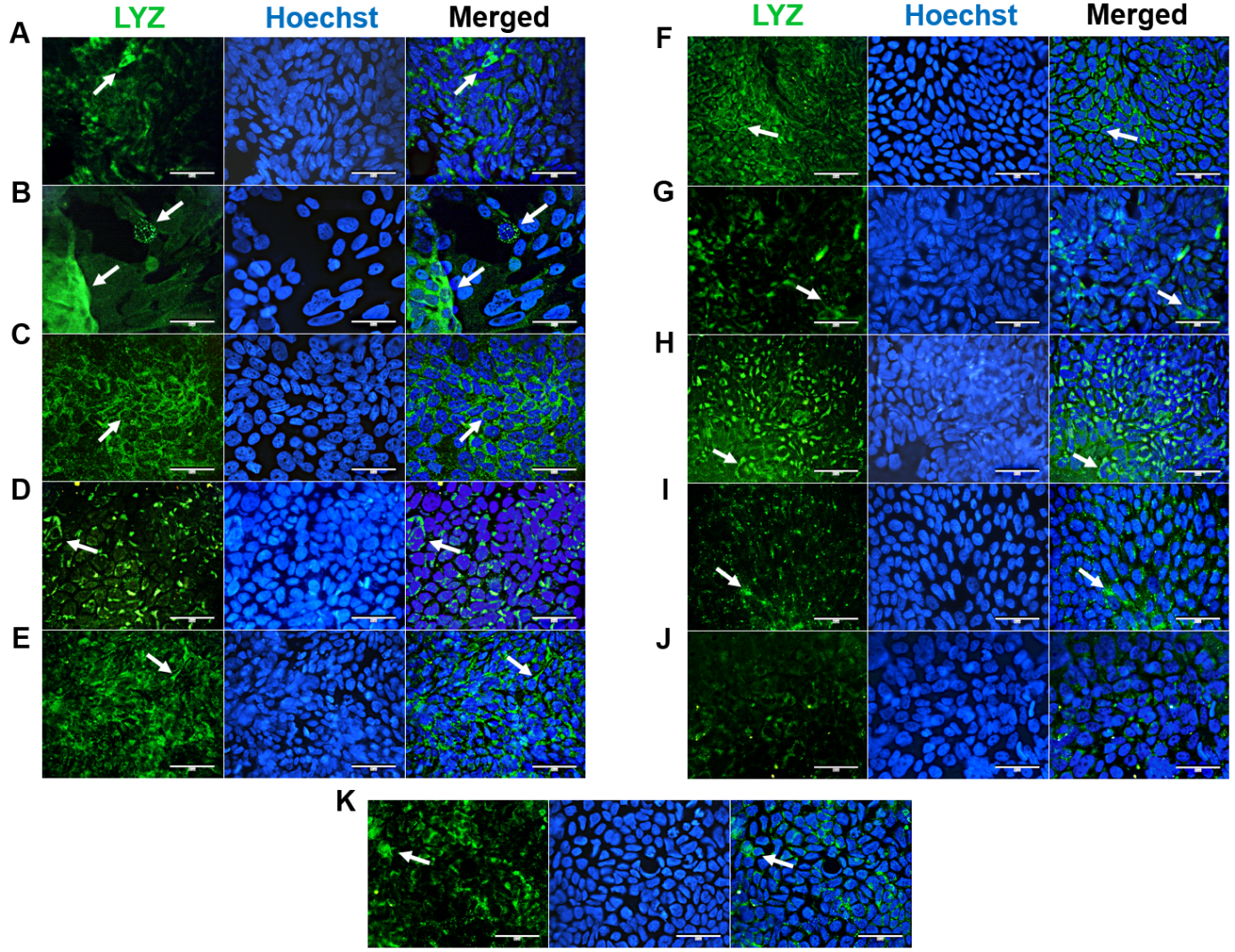


**Figure S2.** Immunofluorescence analysis of LYZ protein localization. (**A**) HROC32, (**B**) HROC43, (**C**) HROC60, (**D**) HROC80 T1 M1, (**E**) HROC126, (**F**) HROC159 T2 M4, (**G**) HROC183 T0 M2, (**H**) HROC217 T1 M2, (**I**) HROC239 T0 M1, (**J**) HROC383 and (**K**) Caco-2. Cells were stained for localization of LYZ by direct immunofluorescence. Arrowheads illustrate representative areas of significant fluorescent staining patterns indicative of PCs. Bars = 50 µm.


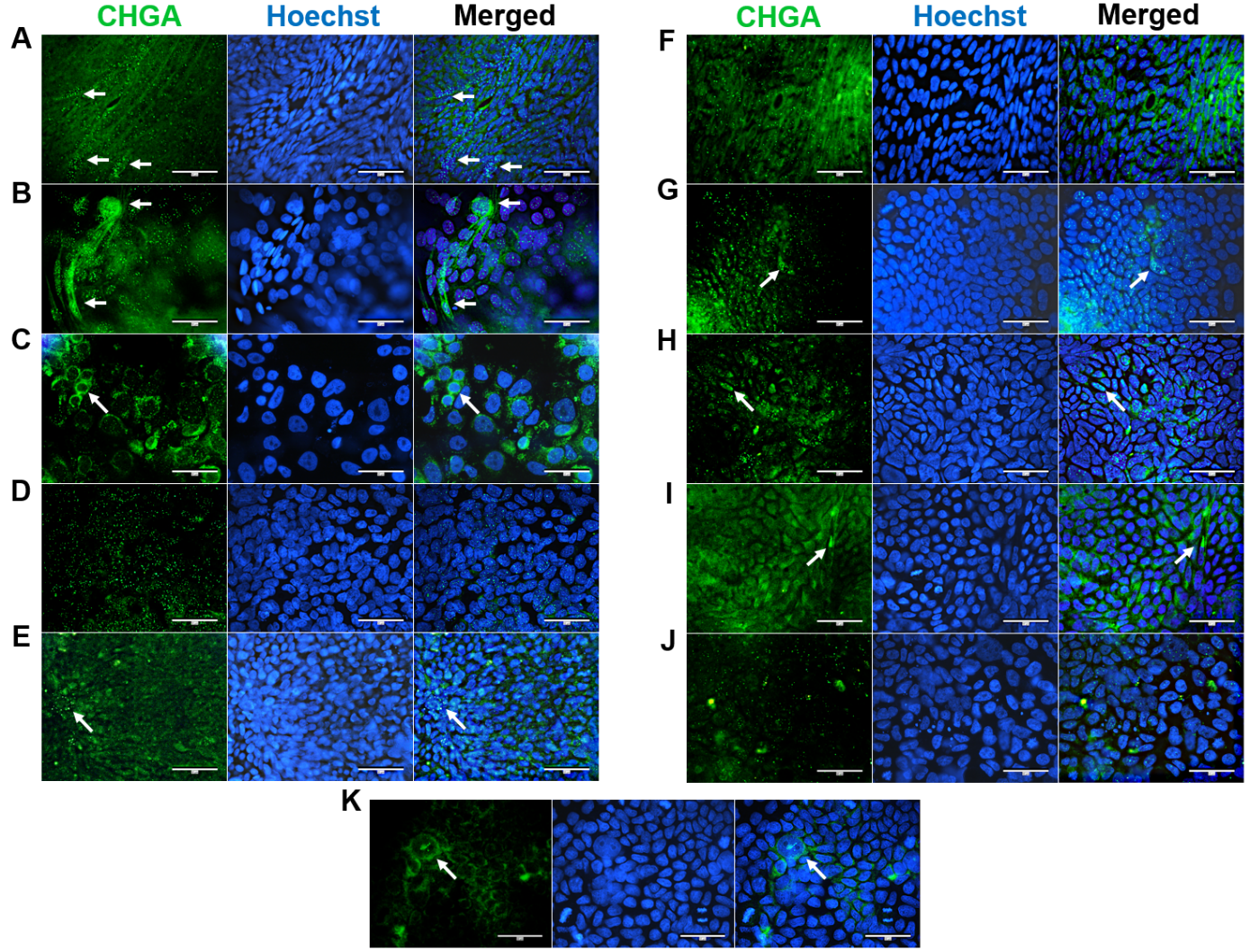


**Figure S3.** Immunofluorescence analysis of CHGA protein localization. (**A**) HROC32, (**B**) HROC43, (**C**) HROC60, (**D**) HROC80 T1 M1, (**E**) HROC126, (**F**) HROC159 T2 M4, (**G**) HROC183 T0 M2, (**H**) HROC217 T1 M2, (**I**) HROC239 T0 M1, (**J**) HROC383 and (**K**) Caco-2. Cells were stained for localization of CHGA by direct immunofluorescence. Arrowheads illustrate representative areas of significant fluorescent staining patterns indicative of EECs. Bars = 50 µm.
